# ACE2 expression is regulated by AhR in SARS-CoV-2-infected macaques

**DOI:** 10.1038/s41423-021-00672-1

**Published:** 2021-04-01

**Authors:** Jiadi Lv, Pin Yu, Zhenfeng Wang, Wei Deng, Linlin Bao, Jiangning Liu, Fengli Li, Qiangqiang Zhu, Nannan Zhou, Qi Lv, Guanpeng Wang, Shunyi Wang, Yabo Zhou, Jiangping Song, Wei-Min Tong, Yuying Liu, Chuan Qin, Bo Huang

**Affiliations:** 1 Department of Immunology and National Key Laboratory of Medical Molecular Biology, Institute of Basic Medical Sciences, Chinese Academy of Medical Sciences (CAMS) and Peking Union Medical College, Beijing, China; 2 NHC Key Laboratory of Human Disease Comparative Medicine, Beijing Key Laboratory for Animal Models of Emerging and Remerging Infectious Diseases, Institute of Laboratory Animal Science, CAMS and Comparative Medicine Center, Peking Union Medical College, Beijing, China; 3 State Key Laboratory of Cardiovascular Disease, Fuwai Hospital, National Center for Cardiovascular Diseases, CAMS and Peking Union Medical College, Beijing, China; 4 Department of Pathology, Institute of Basic Medical Sciences, CAMS and Peking Union Medical College, Beijing, China; 5 Clinical Immunology Center, CAMS, Beijing, China; 6 Department of Biochemistry and Molecular Biology, Tongji Medical College, Huazhong University of Science and Technology, Wuhan, China

**Keywords:** Viral infection, Infection

During the current outbreak of coronavirus disease 2019 (COVID-19) caused by severe acute respiratory syndrome coronavirus 2 (SARS-CoV-2), more than 115 million people have been infected, and 2.5 million have died.^1,2^ Despite such great harm to human health, the pathogenesis of COVID-19 remains unclear. As the first step in the pathogenetic process, viral entry is mediated by the binding of the SARS-CoV-2 surface spike (S) protein to angiotensin-converting enzyme 2 (ACE2) on host cells, such as lung epithelial cells. As an alternative to S protein-blocking strategies, manipulating host cell ACE2 expression may exert an inhibitory effect on SARS-CoV-2 infection. However, the molecular mechanism regulating ACE2 expression remains unclear.

It has been shown that smoking is able to upregulate the expression of ACE2 in lung cells. In addition, single-cell RNA-seq analysis has shown that key antiviral interferons are involved in the regulation of ACE2 expression.^3^ In addition to activating the classical STAT1 pathway, interferons have been shown to activate the cytoplasmic transcription factor aryl hydrocarbon receptor (AhR) through an indoleamine-2,3-dioxygenase-dependent pathway.^4,5^ Notably, tobacco is also able to activate AhR.^6,7^ This coincidence prompted us to hypothesize that AhR transcriptionally regulates the expression of ACE2 in SARS-CoV-2-infected hosts.

To test the hypothesis that ACE2 is regulated by AhR, we initially focused on the tryptophan metabolite kynurenine (Kyn), which is a typical endogenous ligand that activates AhR.^8^ The human lung epithelial cell line BEAS-2B, which expresses ACE2 and can be infected by SARS-CoV-2,^9^ was used as a model cell line to test the possible effect of Kyn on ACE2 expression. Treating BEAS-2B cells with Kyn effectively stimulated the translocation of AhR from the cytoplasm to the nucleus (Figs. 1a and S1a). In the nucleus, AhR was found to bind to the promoter of the *ACE2* gene, as shown by ChIP-qPCR (Fig. 1b). In addition, we found that ACE2 was upregulated at both the mRNA and protein levels in BEAS-2B cells after treatment with Kyn (Fig. S1c, d); however, this upregulation was abrogated by addition of the AhR inhibitor CH223191 (Figs. 1c and S1d), suggesting that upon its activation, the cytosolic transcription factor AhR can regulate ACE2 expression. Similar results were obtained in isolated murine alveolar epithelial type II cells (Figs. 1a, c and S1b–d). Consistent with these findings, in AhR^−/−^ alveolar epithelial cells, Kyn did not have an effect on ACE2 upregulation (Fig. 1d). The photooxidation product of tryptophan, 6-formylindolo(3,2b)carbazole (FICZ), is an exogenous agonist that can effectively activate AhR.^10^ We found that addition of FICZ also resulted in upregulation of ACE2 expression in BEAS-2B and primary murine alveolar epithelial cells in an AhR-dependent manner (Fig. S1e). Together, these results suggest that the expression of ACE2 is regulated by AhR. Consistent with the in vitro data, AhR was localized in the nucleus in alveolar epithelial cells isolated from Kyn-treated mice (Fig. 1e), accompanied by increased ACE2 expression (Fig. S1f). In addition, the expression of ACE2 in the lung tissues was increased in treated mice (Fig. S1g).

**Fig. 1:**
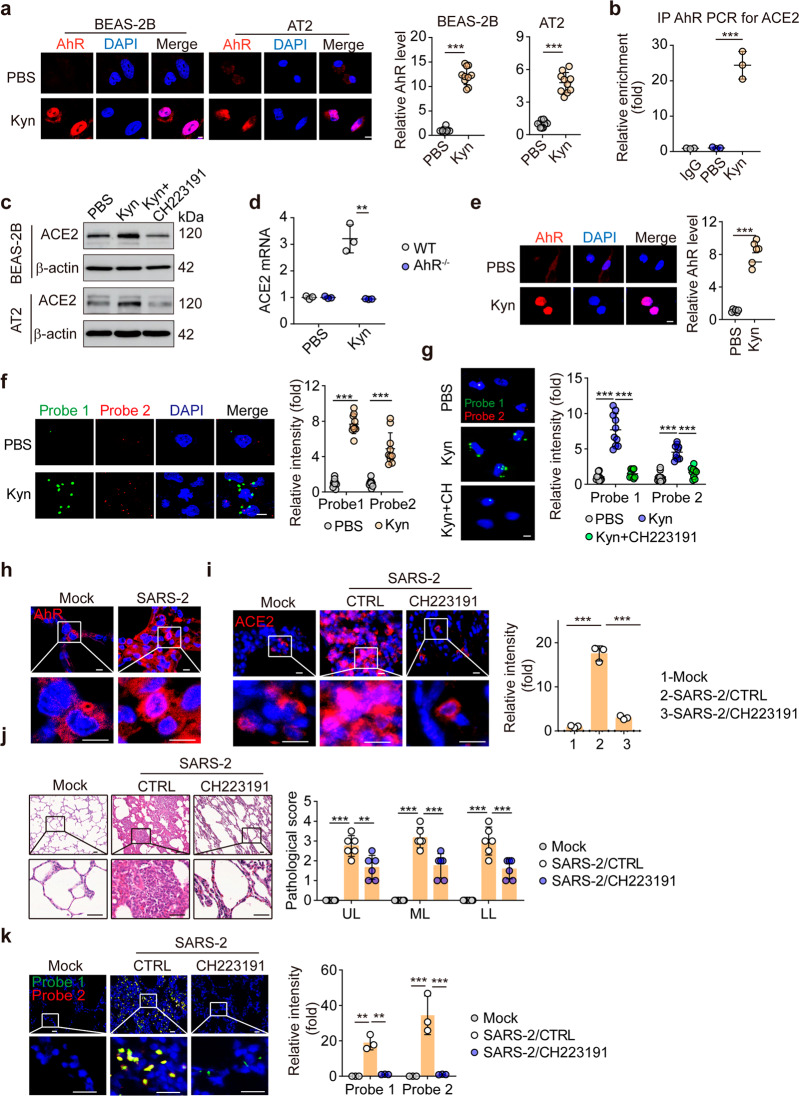
ACE2 expression is regulated by AhR. **a** BEAS-2B cells and primary alveolar epithelial (AT2) cells were treated with PBS or Kyn (0.4 mM) for 48 h. Cells were stained with an anti-AhR antibody and observed under a confocal microscope. Relative AhR expression was calculated from 10 fields per section. Scale bar, 10 μm. **b** The same as **a**, except that BEAS-2B cells were subjected to ChIP-qPCR with the anti-AhR antibody. **c** BEAS-2B cells and AT2 cells were treated with PBS, Kyn (0.4 mM) or Kyn + CH223191 (4 μM) for 48 h. ACE2 expression was determined by western blot analysis. **d** AT2 cells isolated from WT or *AhR*^−/−^ mice were stimulated with Kyn (0.4 mM) for 24 h. ACE2 expression was analyzed by real-time PCR. **e** ICR mice were treated with PBS or Kyn (10 mg/kg) via intratracheal administration once a day for 3 days. Isolated alveolar epithelial cells were stained with the anti-AhR antibody. Scale bar, 10 μm. **f** BEAS-2B cells were pretreated with PBS or Kyn (0.4 mM) for 48 h and were then infected with SARS-CoV-2 at a ratio of 1:1 (TCID_50_:cells) for 48 h. Cells were fixed for RNAscope analysis with SARS-CoV-2-specific probes. The relative intensity was calculated from 10 fields per section. Probe 1 targets the viral sense sequence to evaluate viral distribution (green color); probe 2 targets the viral antisense sequence to indicate viral replication (red color). Scale bar, 10 μm. **g** The same as **f**, except that cells were pretreated with Kyn (0.4 mM) or Kyn + CH223191(4 μM). Scale bar, 10 μm. **h** Macaques were infected with SARS-CoV-2 for 7 days. Lung tissues were stained with the anti-AhR antibody. Scale bar, 10 μm. **i**–**k** Macaques were infected with SARS-CoV-2 and were then treated with vehicle control (CTRL) or CH223191 (1 mg/kg, i.v.) for 7 days. Lung tissues were fixed for staining with the anti-ACE2 antibody (**i**), H&E staining (**j**), and RNAscope analysis (**k**). Three sections from each lung lobe were evaluated per macaque. The representative image shows the viral distribution in damaged lung tissues. Scale bar, 10 μm in **i**, 50 μm in **j**, and 20 μm in **k**. The data are presented as the mean ± SD values. **p < 0.01, ***p < 0.001 by two-tailed *t* test (**a**, **d**, **f**) or one-way ANOVA (**b**, **g**, **i**–**k**)

Given that ACE2 mediates SARS-CoV-2 infection of alveolar epithelial cells, upregulation of ACE2 expression can be expected to enhance SARS-CoV-2 infection and promote cytopathic effects on pneumocytes, thus exacerbating lung pathology. Using RNAscope technology, we found that the viral load was enhanced in BEAS-2B cells pretreated with either Kyn (Fig. 1f). More importantly, the SARS-CoV-2 RNA in the infected cells was active and in a replicating state (Fig. 1f). Consistent with this result, the results of real-time PCR with two pairs of virus-specific primers showed an increased viral load in BEAS-2B cells after 2 or 72 h of SARS-CoV-2 infection compared to that in control cells (Fig. S2a). Then, we blocked the AhR pathway to evaluate the infection of lung epithelial cells by SARS-CoV-2. We found that inhibition of AhR by CH223191 indeed decreased the viral load and suppressed replication in pretreated BEAS-2B cells, as evidenced by both the RNAscope and real-time PCR results (Figs. 1g and S2b).

Finally, we validated the above results in macaques; a COVID-19 model was established in macaques by intratracheal administration of SARS-CoV-2. After 7 days of infection, macaques were euthanized, and lung tissues were collected for immunohistochemical staining. AhR was found to be translocated into the nucleus in the infected lung tissues (Fig. 1h). Consistent with the increased AhR activation, higher ACE2 expression was also found in those lung tissues, as evidenced by immunohistochemical staining (Fig. S3a). In addition, the NP protein level was related to the ACE2 expression level (Fig. S3b). Thus, does decreasing ACE2 expression by inhibiting AhR activity alleviate the pathological damage caused by SARS-CoV-2 infection in the lungs? To test this hypothesis, macaques were infected with SARS-CoV-2 and were then treated with the AhR inhibitor CH223191 for 7 days. Then, the macaques were sacrificed, and the lung tissues were analyzed. Consistent with the decreased expression of ACE2 (Fig. 1i), pathological damage was ameliorated in the infected lungs after CH223191 treatment (Fig. 1j). As expected, the viral load was markedly decreased in the CH223191-treated group, as evidenced by RNAscope, anti-NP immunostaining, and real-time PCR results (Figs. 1k and S3c, d).

In summary, the data in this study clearly show that the transcription factor AhR is able to bind the promoter of the *ACE2* gene, thus promoting ACE2 expression and augmenting the subsequent pathology in SARS-CoV-2-infected lungs.

## Supplementary information

supplymental information

## References

[1] Zhu F-C (2020). Safety, tolerability, and immunogenicity of a recombinant adenovirus type-5 vectored COVID-19 vaccine: a dose-escalation, open-label, non-randomised, first-in-human trial. Lancet.

[2] Wang Y (2020). Remdesivir in adults with severe COVID-19: a randomised, double-blind, placebo-controlled, multicentre trial. Lancet.

[3] Ziegler CGK (2020). SARS-CoV-2 receptor ACE2 is an interferon-stimulated gene in human airway epithelial cells and is detected in specific cell subsets across tissues. Cell.

[4] Liu Y (2017). Blockade of IDO-kynurenine-AhR metabolic circuitry abrogates IFN-gamma-induced immunologic dormancy of tumor-repopulating cells. Nat. Commun..

[5] Liu Y (2018). STAT3/p53 pathway activation disrupts IFN-beta-induced dormancy in tumor-repopulating cells. J. Clin. Invest..

[6] Wang GZ (2019). The Aryl hydrocarbon receptor mediates tobacco-induced PD-L1 expression and is associated with response to immunotherapy. Nat. Commun..

[7] Esser C, Rannug A (2015). The aryl hydrocarbon receptor in barrier organ physiology, immunology, and toxicology. Pharm. Rev..

[8] Liu Y (2018). Tumor-repopulating cells induce PD-1 expression in CD8(+) T cells by transferring kynurenine and AhR activation. Cancer Cell.

[9] Ye R, Liu Z (2020). ACE2 exhibits protective effects against LPS-induced acute lung injury in mice by inhibiting the LPS-TLR4 pathway. Exp. Mol. Pathol..

[10] Rothhammer V, Quintana FJ (2019). The aryl hydrocarbon receptor: an environmental sensor integrating immune responses in health and disease. Nat. Rev. Immunol..

