# Supplementary material for: ACE2 expression is regulated by AhR in SARS-CoV-2-infected macaques
Source: Cell Mol Immunol. 2021 Apr 1;18(5):1308–10. doi: 10.1038/s41423-021-00672-1 (PMC8015744; doi:10.1038/s41423-021-00672-1)
Supplement: Supplementary file 1 — supplymental information [file 41423_2021_672_MOESM1_ESM.docx]

**Supplementary Information**

**ACE2 expression is regulated by AhR in SARS-CoV-2-infected macaques**

Jiadi Lv^1,7^, Pin Yu^2,7^, Zhenfeng Wang^1^, Wei Deng^2^, Linlin Bao^2^, Jiangning Liu^2^, Fengli Li^2^, Qiangqiang Zhu^1^, Nannan Zhou^1^, Qi Lv^2^, Guanpeng Wang^2^, Shunyi Wang^2^, Yabo Zhou^1^, Jiangping Song^3^, Wei-Min Tong^4^, Yuying Liu^1,5*^, Chuan Qin^2*^, Bo Huang^1,5,6,8*^

^1^Department of Immunology & National Key Laboratory of Medical Molecular Biology, Institute of Basic Medical Sciences, Chinese Academy of Medical Sciences (CAMS) & Peking Union Medical College, Beijing 100005, China

^2^NHC Key Laboratory of Human Disease Comparative Medicine, Beijing Key Laboratory for Animal Models of Emerging and Remerging Infectious Diseases, Institute of Laboratory Animal Science, CAMS and Comparative Medicine Center, Peking Union Medical College, Beijing, China.

^3^State Key Laboratory of Cardiovascular Disease, Fuwai Hospital, National Center for Cardiovascular Diseases, CAMS and Peking Union Medical College, Beijing, China

^4^Department of Pathology, Institute of Basic Medical Sciences, CAMS and Peking Union Medical College, Beijing, China

^5^Clinical Immunology Center, CAMS, Beijing 100005, China

^6^Department of Biochemistry & Molecular Biology, Tongji Medical College, Huazhong University of Science & Technology, Wuhan 430030, China

^7^These authors contributed equally

^8^Lead Contact

*Corresponding author: [tjhuangbo@hotmail.com](mailto:tjhuangbo@hotmail.com), [qinchuan@pumc.edu.cn](mailto:qinchuan@pumc.edu.cn) and [13161773902@163.com](mailto:13161773902@163.com)

**Materials and Methods**

**Animals and Cell lines**

Female ICR mice, 6-8 weeks, were purchased from the Center of Medical Experimental Animals of the Chinese Academy of Medical Science (Beijing, China). AhR^-/-^ C57BL/6 mice were presented by Dr. Jun Yan (Third Military Medical University). These animals were maintained in the Animal Facilities of the Chinese Academy of Medical Science under specific pathogen-free conditions. Chinese-origin rhesus macaques (3-4 years old) were obtained from the Institute of Laboratory Animal Science, Peking Union Medical College. Animals studies involving SARS-CoV-2 were performed in an animal biosafety level 3 (BASL3) facility using HEPA-filtered isolators and the procedures were approved by the Institutional Animal Care and Use Committee of the Institute of Laboratory Animal Science, Peking Union Medical College (BLL20001). Murine studies without viral infection were approved by the Animal Care and Use Committee of the Chinese Academy of Medical Science. Normal human bronchial epithelium cell line-BEAS-2B cells were purchased from the China Center for Type Culture Collection (Shanghai, China) and cultured in RPMI1640 medium (Gibco, USA) or DMEM medium (Gibco, USA) with 10% FBS.

**Reagents**

L-Kynurenine was purchased from Sigma-Aldrich (ST, USA). FICZ was purchased from MedChemExpress (NJ, USA) and CH223191 was purchased from Selleck (TX, USA).

**Isolation of primary alveolar epithelial type Ⅱ (AT2) cells**

AT2 cells were isolated from ICR mice or AhR^-/-^ mice as previously reported. Briefly, mice were perfused with 10 ml cold PBS through the right ventricle. Lungs were filled with 2 ml dispase (BD Bioscience, CA, USA) and low gelling temperature agarose (Sigma Aldrich, MO, USA) before lung tissues were incubated with 2 ml dispase in 37ºC for 20 min. Then, lung tissues were rubbed and the slurry was filtered through 70- and 40-μm nylon meshes (JETBIOFIL, China). The cellular suspension was incubated with biotinylated anti-CD45 (Biolegend, clone 30-F11, Cat. 103104), anti-CD16/32 (BD Pharmingen™, clone 2.4G2, Cat. 553143), anti-CD31 (Biolegend, clone MEC13.3, Cat.102504), anti-TER119 (Biolegend, clone TER119, Cat. 116104) and anti-CD104 (Biolegend, clone 346-11A, Cat. 12603) antibodies at 4ºC for 30 min and then Dynabeads® MyOne™ streptavidin T1 magnetic beads (Thermo Fisher Scientific, Cat. 65601) were added to the cell suspension to exclude leukocytes, monocytes/macrophages, NK cells, neutrophils, endothelial cells and erythroid cells. Negative selection of fibroblasts was performed by adherence on non-coated plastic plates. Cell purity was assessed routinely by flow cytometry.

**Western Blotting**

Cells were collected, lysed in M2 lysis buffer and sonicated. The protein concentrations were determined by a BCA kit (Applygen Technologies Inc., China). Then, the protein was run on a SDS-PAGE gel and transferred to nitrocellulose. Nitrocellulose membranes were blocked in 5% bovine serum albumin (BSA) and probed with antibodies at 4̊C overnight: anti-actin (Cell Signaling, Cat No. 3700; 1:1,000) or anti-ACE2 (Abcam, Cat No. ab108252; 1:1,000). Secondary antibodies conjugated to horseradish peroxidase were followed by enhanced chemiluminescence (Thermo fisher, MA). Results were confirmed by at least three independent experiments.

**Real-Time PCR**

Total RNA was extracted from cells using Trizol (Invitrogen) and was transcribed to cDNA by using a high capacity cDNA reverse transcription kit (Applied Biosystems, CA). The primer sequences are shown as follows: *GAPDH,* 5’-ACAACTTTGGTATCGTGGAAGG-3’ (sense) and 5’-GCCATCACGCCA CAGTTTC -3’ (antisense); *ACE2,* 5’- CAAGAGCAAACGGTTGAACAC-3’ (sense) and 5’- CCAGAGCCTCTCATTGTAGTCT-3’ (antisense); *CYP1A1*, 5’-TCGGCCAC GGAGTTTCTTC-3’ (sense) and 5’-GGTCAGCATGTGCCCAATCA-3’ (antisense); *CYP1B1*, 5’-TGAGTGCCGTGTGTTTCGG-3’ (sense) and5’- GTTGCTGAAG TTGCGGTTGAG-3’ (antisense); *Gapdh,* 5’- AGGTCGGTGTGAACGGATTTG-3’ (sense) and 5’-TGTAGACCATGTAGTTGAGGTCA-3’ (antisense); *Ace2,* 5’-TCCA GACTCCGATCATCAAGC-3’ (sense) and 5’-GCTCATGGTGTTCAGAATTG TGT-3’ (antisense); *Cyp1a1*, 5’-GACCCTTA CAAGTATTTGGTCGT-3’ (sense) and 5’-GGTATCCAGAGCCAGTAACCT-3’ (antisense); *Cyp1b1*, 5’-CACCAGCCTTAG TGCAGACAG-3’ (sense) and 5’-GAGGACCACGGTTTCCGTTG-3’ (antisense); SARS-CoV-2 primer1 (*ORF1ab*): 5’-CCCTGTGGGTTTTACACTTAA-3’ (sense) and 5’-ACGATTGTGCATCAGCTGA-3’ (antisense); SARS-CoV-2 primer2 (*Nucleoprotein*): 5’-GGGGAACTTCTCCTGCTAGAAT-3’ (sense) and 5’-CAGACA TTTTGCTCTCAAGCTG-3’ (antisense). Real-time PCR was performed using ABI step-one plus (Applied Biosystems, CA, USA). Values are means ± SD from three independent experiments which were performed in duplicate. Statistical comparisons among groups were performed using a Student’s t-test. Values of all parameters were considered statistically significant at a value of p<0.05.

**Immunofluorescence**

Cells were fixed in 4% paraformaldehyde and permeabilized with 0.2% Triton X-100. Fixed cells were blocked in 5% BSA and incubated with anti-AhR (GeneTex, Cat No. GTX129013, 1:200) antibody or anti-ACE2 (Abcam, Cat No. ab108252, 1:200) antibody at 4̊C overnight, cells were washed and incubated with secondary antibodies for 1 hr at room temperature. Finally, the slides were counterstained with DAPI and mounted for confocal analysis. The intensity of immunofluorescence was analyzed by Image J 9.0 software.

**Histological and immunohistochemical staining**

The lung tisses from mice or rhesus macaques were fixed in 10% formalin, embedded in paraffin and sectioned for H&E staining. According to morphological changes after SARS-CoV-2 infection, the lung tissues were graded as mild (1), moderate (2), severe (3) or life-threatening (4). An expert in pathology who was blinded to the experiment gave a score based on the inflammatory cell infiltration, parenchymal pneumonia, alveolar hemorrhage and bronchiolar/bronchial luminal or alveolar exudate. Immunohistochemical staining was performed according to a protocol as previously described.^34^ Briefly, the sections of paraffin embedded tissues were incubated with anti-ACE2 (Abcam, Cat No. ab108252, 1:1,000) antibody or anti-SARS nucleocapsid protein (Abcam, Cat. Ab273434, 1:1,000) antibody; anti-AhR antibody (GeneTex, Cat No. GTX113124, 1:1,000 dilution) at 4̊C overnight. Afterwards, slides were sequentially incubated with two HRP-conjugated secondary antibodies for 1 hr at room temprature. For fluorescent staining, the slides were incubated with PANO Reagent PPD520 or PDD570 using the PANO 4-plex IHC Kit (Panovue, China) according to the manufacturer’s instructions, followed by conterstaining with DAPI (Thermo, USA) and finally mounting for confocal analysis. The stained lung sections were scanned and digitalized utilizing a TissueFaxs Plus System coupled onto a Zeiss Axio Imager Z2 microscope and Nikon A1 confocal microscope. The intensity of positive staining was analyzed by Image J 9.0 software.

**ChIP-qPCR**

ChIP-qPCR was performed by using a MAGnityTM Chromatin Immunoprecipitation System (Invitrogen, USA) according to the manufacturer’s protocol. In brief, BEAS-2B cells were crosslinked and chromatin was extracted and sheared. Samples were immunoprecipitated with anti-AhR antibody (Cell Signaling, Cat No. 83200). The primer sequences used for ChIP-qPCR are shown as follows: *ACE2*, 5’- CGGGTGCGGTGG CTCATGCC -3’ (sense) and 5’- CCGCCTCCCAGGTTCACGCC -3’ (antisense). The results were from three independent experiments followed by normalization to input signals and shown as mean ± SD.

**RNA *in situ* hybridization (RNA-ISH)**

RNA-ISH was performed on BEAS-2B cells grown on glass coverslips or paraffin-embedded 5 μm lung tissue sections using the RNAscope Multiplex Fluorescent Assay v2 according to the manufacturer’s instructions (Advanced Cell Diagnostics, USA). Briefly, Cells were fixed in 4% paraformaldehyde and permeabilized with 0.1 % Triton X-100, incubated with hydrogen peroxide at RT for 10 min and 1:15 diluted Protease Ⅲ at RT for 10 min. Lung tissue sections were deparaffinized with xylene and rehydrated with graded ethanol, incubated with hydrogen peroxide, and then boiled for 15 min in Target Retrieval buffer, followed incubated with Protease Plus for 15 min at 40̊C. Slides were hybridized with SARS-CoV-2 probes in a hybridization oven at 40̊C for 2 hr, and the fluorescent signals were amplified according to manufacturer’s protocol. The BEAS-2B cells grown on glass coverslips and stained lung sections were scanned and digitalized utilizing a TissueFaxs Plus System coupled onto a Zeiss Axio Imager Z2 microscope. The intensity of immunofluorescence and positive cell rate were analyzed by Image J 9.0 software.

**Animal experiments and treatment protocol**

Rhesus macaques (3 to 4 kg, 3 to 4 years of age) were infected with SARS-CoV-2 (10^6^ TCID_50_) by intratracheal administration, and then treated with control vehicle (CTRL) or CH223191 (1 mg/kg, i.v.) once a day for 7 days (n = 3 macaques/group). After 7 days of treatment, macaques were euthanized and lung tissues were collected for real-time PCR assay and histological and immunohistochemical staining.

**Quantification and Statistical Analysis**

All experiments were performed at least three times. Results are expressed as mean ± SD as indicated and analyzed by two-tailed Student's t-test or One-way ANOVA followed by Boferroni’s test. The p value < 0.05 was considered statistically significant. The analysis was conducted using the Graphpad 8.0 software.

**Figure legends**


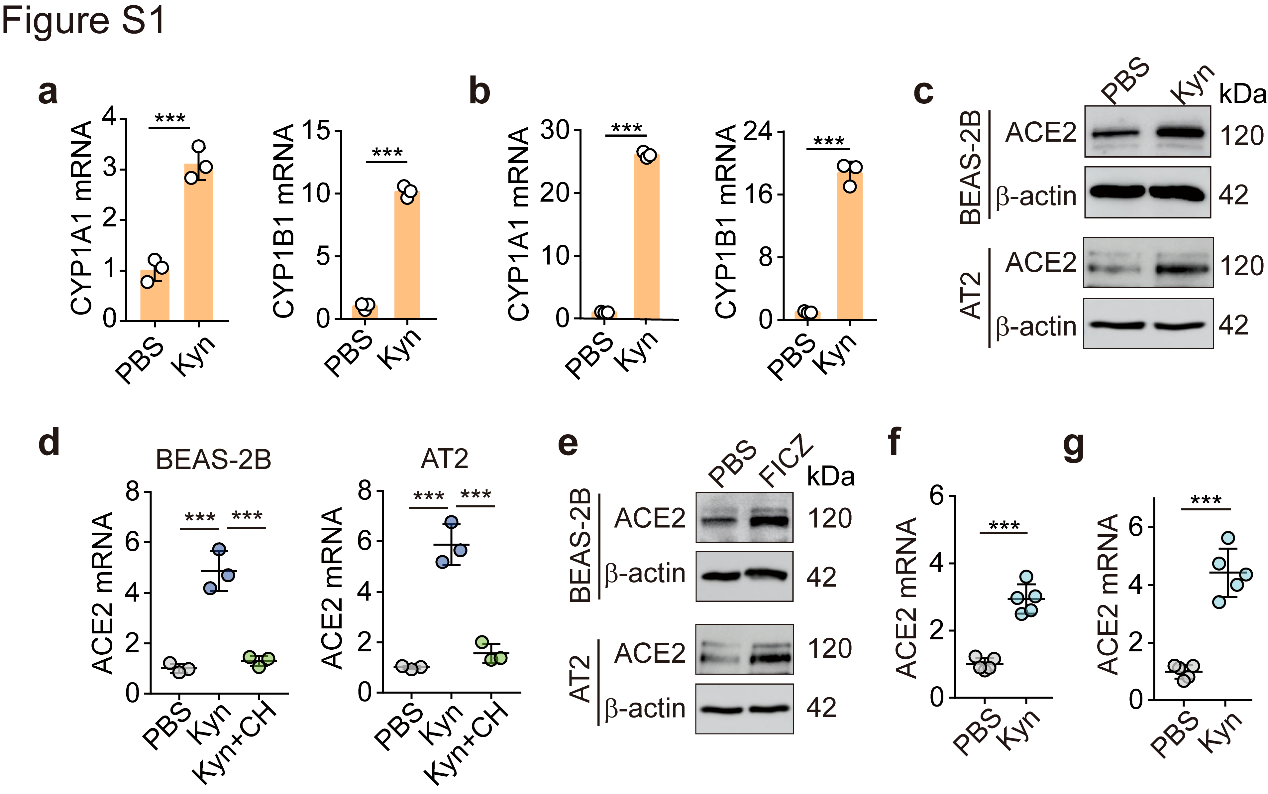


**Fig. S1** Kyn upregulate ACE2 expression. **a, b** BEAS-2B cells (a) or AT2 cells (b) were treated with IFN-β (1 ng/ml), IFN-γ (10 ng/ml) or Kyn (0.4 mM) for 48 hr. The expression of *CYP1A1* and *CYP1B1* were determined by real-time PCR. **c** BEAS-2B cells or primary alveolar epithelial (AT2) cells were treated with PBS or Kyn (0.4 mM) for 48 hr. ACE2 expression was determined by western blot. **d** BEAS-2B cells or AT2 cells were treated with Kyn (0.4 mM) or Kyn + CH223191 (4 μM) for 24 hr. The expression of ACE2 was measured by real-time PCR. **e** The same as (**c**), except that cells were stimulated with FICZ (1 μM). ACE2 expression was determined by western blot. **f, g** ICR mice were treated with Kyn (10 mg/kg) by intratracheal administration once a day for 3 days. the expression of ACE2 from the isolated alveolar epithelial cells (**f**) or the lung tissues (**g**) was determined by real-time PCR. n = 5 mice from every group. The data represent mean ± SD. *** p<0.001, by one-way ANOVA (**d**) or two-tailed *t* test (**a, b, f** and **g**).


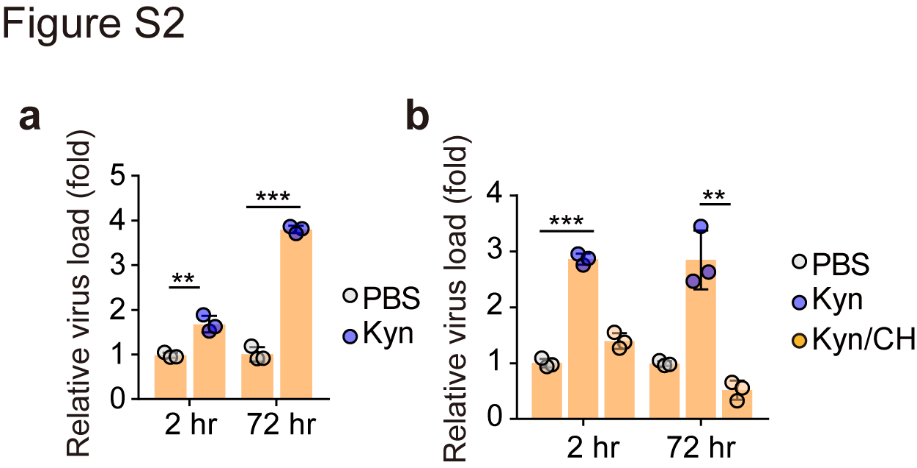


**Fig. S2** AhR inhibitor prevents the upregulation of ACE2 induced by Kyn. **a, b** BEAS-2B cells were pretreated with Kyn (0.4 mM), or Kyn + CH223191 (4 μM) for 48 hr, and then infected with SARS-CoV-2 at the ratio of 1:1 (TCID50 : cells) for 2 hr or 72 hr. Viral load was analyzed by real-time PCR with specific primer 2. The data represent mean ± SD. ** p<0.01, *** p<0.001, by one-way ANOVA (**b**) or two-tailed *t* test (**a**).


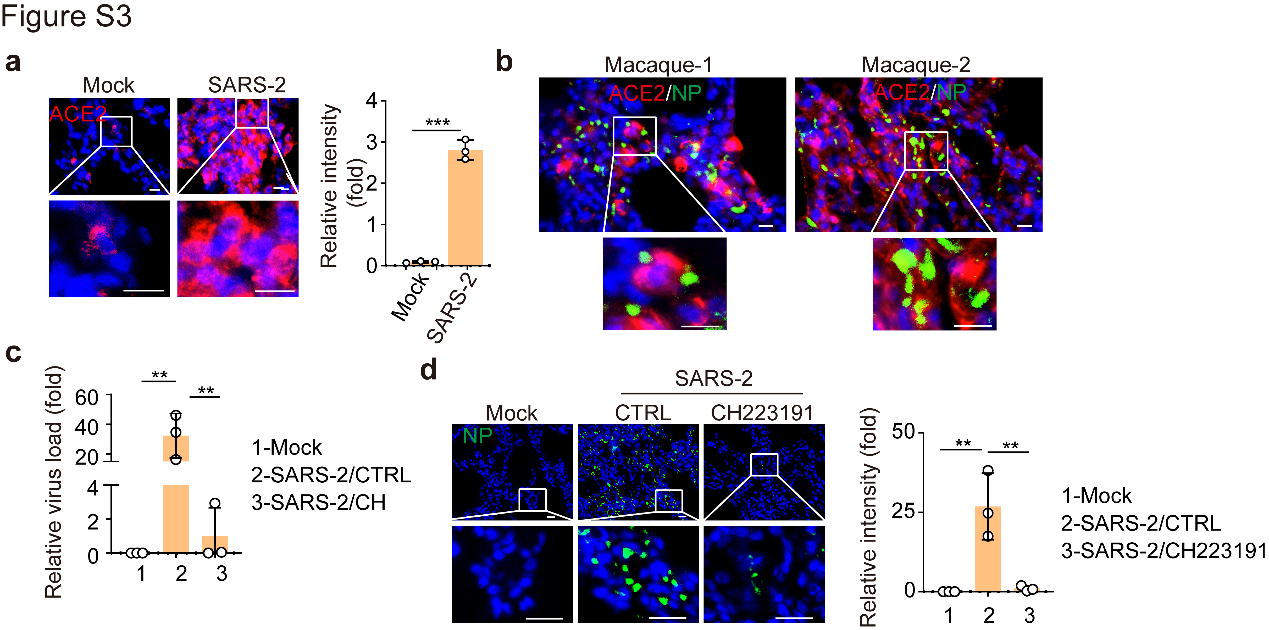


**Fig. S3** AhR blockade alleviated the virus load in the lung tissues of macaques infected by SARS-CoV-2. **a, b** Macaques were infected with SARS-CoV-2 for 7 days. Lung tissues were fixed and stained with anti-ACE2 antibody (**a**, red color) or anti-NP antibody (**b**, green color) or (n = 3 macaques for each group). **c** Macaques were infected with SARS-CoV-2, and then treated with control vehicle (CTRL) or CH223191 (1 mg/kg, i.v.) for 7 days. The lung tissues were used for real-time PCR. **d** The same as (**c**), except that the lung tissues were stained with anti-NP antibody. Scale bar, 10 μm. The data represent mean ± SD. ** p<0.01, *** p<0.001, by one-way ANOVA (**c** and **d**) or two-tailed *t* test (**a**).
